# Role of neutrophil-lymphocyte ratio as a prognostic marker for type 2 diabetic nephropathy among Indians

**DOI:** 10.6026/97320630019375

**Published:** 2023-04-30

**Authors:** Murugan Subramani, Mudali Anbarasan, Deepalatha Shanmugam, Lakshmi Narayanan Muthumani, Pradeep Vasudevan

**Affiliations:** 1 Department of General Medicine, K.A.P Viswanatham Govt Medical College, Trichy - 17; 2 Department of Microbiology,Thanjavur Medical College, Thanjavur, India

**Keywords:** Diabetic nephropathy, urinary albumin-to-creatinine ratio, neutrophil-to-lymphocyte ratio

## Abstract

Diabetic nephropathy/diabetic kidney disease (DKD) is one of the leading causes of renal failure. Early identification of the development or progression of diabetic nephropathy using appropriate screening and diagnostic tools is very important in order to provide timely and proper management. Inflammation plays a crucial role in development and progression of diabetic nephropathy. The aim of this study was to evaluate the relationship of inflammatory markers (neutrophil-to-lymphocyte ratio-NLR) as an early indicator to prevent the progression of diabetic kidney disease. A total of 158 patients with type 2 diabetes mellitus were distributed into three groups according urinary albumin-to-creatinine ratio. Levels of inflammatory markers neutrophil-to-lymphocyte ratio was recorded and compared among the three groups. Significant differences were detected between the groups in terms of neutrophil-to-lymphocyte ratio (p = 0.000).Characteristic curve analysis of inflammatory markers and microalbuminuria prediction demonstrated an area under curve (AUC) of 0.869 for neutrophil-to-lymphocyte ratio (p = 0.000). A NLR cut-off point of 2.2 has 72.3 % sensitivity and 78.1 % specificity, which suggested sufficient accuracy. Increased neutrophil-to-lymphocyte ratio was significantly correlated with diabetic nephropathy progression and increased neutrophil-to-lymphocyte ratio can be considered as an early indicator and a prognostic risk marker of diabetic nephropathy.

## Background:

Diabetes Mellitus (DM) is a serious threat to global health with an increasing prevalence and incidence rates. The number of people who had DM was 463 million in 2019. It is estimated that this number will have reached 700 million by 2045 according to International Diabetes Federation (IDF) [1]. DM is a chronic metabolic disease characterized by high sugar levels (hyperglycemia) due to impairment of insulin secretion, cellular resistance to insulin or both [2]. DM is classified into two major types; type-1 in which the pancreas is unable to produce insulin and type -2 in which insulin secretion is not adequate or the body is unable to respond to it proficiently [3].

DM causes serious complications such as diabetic nephropathy (DN), diabetic retinopathy, and diabetic neuropathy (microvascular complications) in addition to stroke, cardiovascular diseases (CVDs), and peripheral vascular diseases (macro vascular complications) [4, 5].DN or diabetic kidney disease is a syndrome described by the presence of pathological amounts of proteinuria, diabetic glomerular lesions, and decrease of glomerular filtration rate (GFR) in diabetic patients [6]. DN is now the most common reason of chronic kidney disease (CKD) [7], yet DN pathogenesis is not fully understood. Both types of diabetes can cause chronic kidney disease and eventually end – stage renal disease (ESRD) [9]. However, the prevalence of type 2 diabetes is much higher than type 1, often patients with ESRD have type 2 diabetes [10].

An increase in urinary albumin excretion is a clinical manifestation for DN, starting from micro albuminuria to macro albuminuria and eventually ESRD [8, 9]. Current diagnosis of DN is depended on albuminuria as a biomarker [11]. However, its diagnostic value in early-stage DN is limited because renal injury commonly precedes urinary albumin secretion [6].Inflammation plays a crucial role in development and progression of DN, as many inflammatory cytokines such as interleukin-1 (IL-1), interleukin-6 (IL-6),interleukin-8 (IL-18), and tumor necrosis factor α (TNF-α) contribute in the pathogenesis of DN [12]. However, the measurement of these inflammatory markers is not used in daily clinical practice because of their costs and technical difficulties in application [13].

In this respect, neutrophil-to-lymphocyte ratio (NLR) has emerged as a novel alternative marker [11]. Increased NLR and PLR were significantly correlated with DN, and high NLR and PLR may be served as a predictor and a prognostic risk marker of DN. These parameters are easy to calculate in the laboratory. NLR and PLR tests are simple, cost-effective, and done routinely. They can be beneficial as alternative markers for inflammation [14]. NLR has been proposed as surrogate markers for endothelial dysfunction and inflammation. Early and timely assessment with simple diagnostic modalities is beneficial for identifying and managing diabetic nephropathy. Many investigators are now focusing on early biomarkers to predict kidney damage beyond albuminuria [15].To improve the lives of people with diabetic nephropathy and to reduce the impact on society, early identification of the development or progression of diabetic nephropathy using appropriate screening and diagnostic tools is very important in order to provide timely and proper management. The aim of this study was to evaluate if NLR can be used as early predictors and prognostic risk markers of DN and to lower the development of the complications upon uncontrolled diabetic patients, which will help physicians and diabetologist to identify the disease progression early and provide an appropriate treatment. Therefore, there is an unmet need for reliable surrogate biomarkers to monitor the onset and progression of early changes and facilitate drug discovery.

Depending on the clinical setting involved, identifying and monitoring diabetic nephropathy primarily involves two diagnostic modalities: assessment of kidney function in terms of estimated glomerular filtration rate (eGFR) and estimation of kidney damage in terms of albuminuria [16].These methods are now used worldwide as clinical markers of diabetic nephropathy in real practice. Moreover, these markers help to decide whether or not to apply early therapeutic techniques and provide information to assess the risks of CVD and ESRD in diabetic nephropathy. However, these markers have several limitations with regard to identifying and monitoring diabetic nephropathy. [15]. Recognition of these limitations and the efforts to investigate better and new biomarkers are essential for the efficacious management of diabetic nephropathy.

## Materials and methods:

## Study design:

Observational cross sectional analytical study.

## Study Centre:

Department of General Medicine, Mahatma Gandhi Memorial Government Hospital, Tiruchirappalli.

## Duration of study:

November 2021 To April 2022.

## Study population:

Patients with Type 2 diabetes mellitus attending department of General Medicine, Mahatma Gandhi Memorial Government Hospital.

a) Inclusion criteria: All patients with diagnosed Type 2 Diabetes Mellitus.

b) Exclusion Criteria:

1) Patients with type 1 DM

2) Patient with systemic hypertension

3) Patient with chronic kidney disease

4) Patient with chronic liver disease

5) Patients with infection

6) Patient on anti-inflammatory drugs, systemic steroids

7) Patient having disease affecting urinary protein excretion as nephrotic syndrome,urolithiasis, renal insufficiency, renal artery stenosis, UTI.

## Sample Size:

158 patients with type 2 diabetes mellitus.

## Data collection and methods:

After getting informed consent from patients who have type 2 DM, a proforma consisting information on patient id, name of the patient, age, gender, duration of diabetes was used.

## Procedure:

Patients attending the diabetic outpatient department were subjected to tests such as complete blood count, renal function test, urine routine microscopy, urinePCR, 24 hours urine protein, fasting blood sugar, post prandial blood sugar and HbA1c. Albuminuria was tested by dipstick method, urinary PCR, 24 hr urine protein was also assessed. Complete blood count was estimated by automated blood counter, Glycemic index was assessed by fasting blood sugar and post prandial blood sugar by glucose oxidase method in auto analyzer.

Evaluation of diabetic nephropathy was done by examining urine for albuminuria. According to the American diabetes association and Mogensen diabetic nephropathy diagnostic criteria:

[1] Group 1- Normal -albuminuria (urinary albumin-tocreatinine ratio <30 mg/g)

[2] Group 2 - Microalbuminuria (urinary albumin-tocreatinine ratio = 30-300 mg/g)

[3] Group 3 - Macro-albuminuria (urinary albumin-tocreatinine ratio ≥300 mg/g). Levels of inflammatory markers neutrophil-to-lymphocyte ratio was recorded and compared among the three groups.

1) Ethical approval: Ethical approval for the study was obtained from the institutional ethics committee of K.A.P. Viswanatham Government medical college, Tiruchirappalli. Written informed consent was obtained from all study participants.

2) Statistical analysis: The collected data was entered in Microsoft Excel and transferred to SPSS software for analysis. Statistical difference between two proportions was analyzed using chi-square test. To analyse the difference in mean between 2 groups, independent t test was done. Binary logistic regression was used for multivariate analysis. For all tests of statistical significance, p value of <0.05 was taken as significant.

## Results and Discussion:

A total of 158 patients with type 2 diabetic patients were enrolled in this study. The patients distributed into three groups according to their level of albumin-to-creatinine ratio, designated as normoalbuminuria (N = 67, 42.4%), microalbuminuria (N = 50, 31.6%) and macro albuminuria (N = 41, 25.9%). No significant difference was observed between the groups with regard to BMI, serum albumin, monocyte count, and RBC. However, there were significant differences among the three groups regarding age (p = 0.000), duration of diabetes (p = 0.000), HbA1c (p = 0.000), blood pressure (p = 0.000), serum urea (p =0.000), serum creatinine (p = 0.000), GFR (p = 0.000), total WBC (p =0.000), absolute neutrophil count (p = 0.000), and inflammatory markers NLR (p = 0.000). Univariate analysis (Pearson) found significant correlation between NLR and duration of diabetes, HbA1c, blood pressure, urea, creatinine, GFR, albumin/creatinine ratio, WBC, PLR, and CRP.Table 1

Receiver operating characteristic curve analysis of NLR for microalbuminuria prediction found an area under curve of 0.869 for NLR (confidence interval: 0.813-0.926, p = 0.000). A NLR cut-off point of 2.2 has 72.3 % sensitivity and 78.1 % specificity, which suggest sufficient accuracy. ROC curve analysis and selected cut off points for NLR is presented in Table 2, Table 3.

The main purpose of this study was to investigate and evaluate the predictive value of NLR for DN in type 2 diabetic patients. The sample consisted of patients with type 2 diabetes who were divided into three groups according to their albumin-to-creatinine ratio. Levels of inflammatory markers (NLR) and other parameters were compared among the three groups. Results indicated that the NLR values were significantly higher in the diabetic patients with macro albuminuria than in those with microalbuminuria and those without albuminuria. DN is a common severe complication in patients with diabetes, but its exact pathogenesis remains unclear [17].Although microalbuminuria is a strong marker for DN diagnosis and progression, glomerular damage is considered as early sign of DN and precedes the appearance of microalbuminuria [18].

It is known that a cascade of pathological events (glomerular damage gives rise to proteinuria, followed by progressive renal damage, fibrosis, inflammation, and finally loss of functional nephrons) is involved in the development and progression of DN. Accumulated evidences have demonstrated that chronic inflammation plays a key role in the development of DM-associated complications [13]. Several studies have associated DN with chronic inflammation, as various inflammatory molecules such as adipokines, chemokines, adhesion molecules, and cytokines could contribute in the development of DN [19, 20]. Thus, evaluating the associations between the NLR level and different diabetic complications is important. NLR was recognized as a predictive marker in cardiovascular diseases (such as coronary artery disease, acute coronary syndromes, and heart failure) and in several types of cancer.

Wan *et al.*. reported that a higher NLR level was associated with an increased prevalence of cardiovascular and cerebrovascular diseases, and diabetic kidney disease in diabetic adults [13].A study by Ozturk *et al.*. showed that NLR is an independent predictor for microvascular complications in geriatric diabetic subjects [21]. Moursy *et al*. indicated that NLR is not only an efficient and stable index of inflammation, but also a crucial predictor for the presence of microvascular diabetic complications in Egyptian patients with type-2 diabetes [22].

In reference to glycemic parameters, there were significant differences among the groups, HbA1c values were higher than 7% in all study groups, and this may have indicated poor glycemic control in type 2 diabetic patients. It can also be considered as a disease-monitoring tool during the follow-up of patients with diabetes. Moreover, NLR is influenced by genetic and non-genetic factors (sex, age, seasonal conditions, lifestyle and diseases) [23]. In the present study, the prevalence of diabetic kidney disease was 74%, which was much higher than the prevalence reported by Khandare *et al*. [24] where the prevalence of diabetic nephropathy among type 2 diabetes patients was 48.7%.

So that, this study has some limitations namely, small number of patients (n = 158), retrospective design, the lack of accessibility to some data such as, cholesterol, 24-hour urinary albumin excretion, etc. In the current study, neutrophil lymphocyte ratio was significantly high among patients with DKD compared to their counterpart patients without DKD. Similar finding was reported by Khandare *et al.*. [24] where mean NLR was significantly high in patients with DN compared to patients without DN.In a metaanalysis done by Liu *et al.*. [23] , neutrophil lymphocyte ratio was found to have significant association with degree of diabetic nephropathy. Dudani *et al.*. [25] in India Neutrophil lymphocyte ratio was found to be significantly higher among patients with diabetes compared to the control group, which proves that there is a role of inflammation in occurrence of diabetes. In their study, Wan H *et al.*. [13] reported that presence of diabetic kidney disease was very high among patients with higher levels of neutrophil lymphocyte ratio. In our study, we did not find any statistically significant association between age and NLR. Wan H found a significant association between age and neutrophil lymphocyte ratio. In our study, proportion of patients with DKD was high in patients with poor glycemic control. In contrast to this, Khandare *eta*l. [24] reported that HbA1c did not have significant association with presence or absence of DN. Both low eGFR and albuminuria are associated independently with higher rates of mortality and progression to ESRD in KEEP participants with diabetes. A significant synergistic interaction between lower eGFR and greater degree of albuminuria occurs in this group, such that the risk of mortality and progression to ESRD is amplified when both factors are present [26].

In the current, study NLR level increased with increase in duration of diabetes. Wan *et al.*. [13] also reported that patients with more duration of diabetes had higher neutrophil lymphocyte ratio. In our study patients with poor glycemic control with HbA1c level ≥6.5 had high NLR. Similar findings were reported by Wan *et al.*. [13] where Neutrophil lymphocyte ratio was found to increase with increase in HbA1c levels. Sefil *et al.*. [27] also found that neutrophil count was significantly higher among patients with high HbA1c.In consistent with these findings Akin *et al.*. [28] and Umarani *et al.*. [29] also found that Neutrophil lymphocyte ratio was significantly higher among patients with poor glycemic control. In our study, we found that there was proportionate increase in NLR with increase in 24 hours urine albumin excretion. Similar to our finding, Kahraman *et al.*. [30] found a linear relationship between NLR and 24 hours urine albumin excretion. In our study, there was a linear positive correlation between serum creatinine and NLR. Kahraman *et al.*. [30] reported that there was a significant positive correlation between NLR and serum creatinine in their study. In the present study, patients with low eGFR had high NLR levels. Similar to this finding, Wan *et al.*. [13] also reported that there was a gradient reduction in estimated glomerular filtration rate with increase in neutrophils lymphocytes ratio. In the present study, with multivariate analysis we found that neutrophil lymphocyte ratio was an independent predictor of diabetic kidney disease when compared to age of the patients, their gender, and duration of diabetes, serum creatinine level and eGFR. Similarly, Mohammad *et al.* also reported that neutrophil lymphocyte ratio was found to be the independent predictor of albuminuria among patients with type 2 diabetes mellitus when compared to duration of diabetes, HbA1c level and estimated glomerular filtration rate.

## Conclusion:

NLR proportionately increased with increase in 24 hours urine albumin excretion. Increased neutrophil-to-lymphocyte ratio was significantly correlated with diabetic nephropathy progression and increased neutrophil-to-lymphocyte ratio can be considered as an early indicator and a prognostic risk marker of diabetic nephropathy NLR was found to be an independent predictor of albuminuria. These parameters are easy to calculate in the laboratory. NLR tests are simple, cost-effective, and done routinely and they can be beneficial as alternative markers for inflammation.

## Figures and Tables

**Table 1:** Summaries of results regarding the demographic, clinical, and laboratory characteristics of the study groups

**Parameter**	**Normo-albuminuria (N = 67)**	**Microalbuminuria (N = 50)**	**Macroalbuminuria (N = 43)**	**P-value**
	**Mean±Std.Dev**	**Mean±Std.Dev**	**Mean±Std.Dev**	
Age (year)	54±10	58±7	61±6	0
Male/Female (N)	39/28	29/21	24/17	NS
Smoking (Yes/No)	27/40	21/29	16/25	NS
BMI (kg/m2)	27.66±2.4	27.27±2.37	27.5±1.39	NS
Duration of Diabetes (year)	6.7±1.48	10.32±1.85	12±1.88	0
HbA1c (%)	7.95±1.49	8.55±1.29	8.92±1.35	0.002
Systolic Blood Pressure (mmHg)	12.16±0.88	13±0.76	14.37±1.11	0
Diastolic Blood Pressure (mmHg)	8±0	8.44±0.76	8.93±0.82	0
Serum urea (mg/dl)	23.91±8.14	40.14±12.54	41.95±7.83	0
Serum creatinine (mg/dl)	0.73±0.08	0.87±0.14	1.04±0.18	0
Serum albumin (g/dl)	4.25±0.33	4.23±0.37	4.16±0.39	NS
GFR (ml/min/1.73m2)	107.7±14.75	87.04±12.2	70.32±7.23	0
Absolute Neutrophil count (/µl)	4145.23±1318.34	5183.63±1457.24	5961.7±1198.02	0
Absolute Lymphocyte count (/µl)	2448.04±638.35	2290.26±527.6	1992.93±446.27	0
NLR	1.73±0.47	2.3±0.58	3.03±0.46	0
Albumin/creatinine (mg/g)	9.90±3.93	103.71±65.15	530.40±168.49	0

**Table 2:** Pearson's correlation analysis of NLR

**Variable**	**NLR**	
	**r**	**P value**
BMI	-0.036	0.654
Duration of Diabetes	0.537	0
HbA1c	0.343	0
Systolic Blood Pressure	0.431	0
Diastolic Blood Pressure	0.41	0
Serum urea	0.407	0
Serum creatinine	0.537	0
Serum albumin	-0.036	0.65
Albumin/Creatinine	0.659	0
GFR	-0.626	0
WBC	0.431	0
Neutrophil count	0.66	0
Lymphocyte count	-0.418	0
RBC	-0.059	0.46
Hb	-0.29	0
PLT	0.153	0.055
PLR	0.483	0
CRP	0.653	0

**Table 3:** Receiver operating characteristic (ROC) curve analysis for prediction of microalbuminuria using NLR cut-off values

	**Area under curve**	**cut off**	**Sensitivity (%)**	**Specificity (%)**
NLR	0.869	2.2	72.3	78.1
